# Adult Ascaris Worm Passing from the Mouth

**DOI:** 10.4269/ajtmh.2011.11-0258

**Published:** 2011-09-01

**Authors:** Jacques Margery, Abdourahmane Niang

**Affiliations:** Service de Pneumologie, Hôpital d'Instruction des Armées Percy, Clamart-France; Service de Pneumologie, Hôpital Principal, Dakar-Sénégal

*Ascaris lumbricoides* is one of the most widespread human infections. Adult *Ascaris* worms inhabit the small intestine and themselves cause little disease in their normal habitat, except when there is heavy infection leading to intestinal obstruction. Wandering ascarids may reach abnormal situations when they migrate through the ampulla of Vater causing biliary obstruction with cholangitis and/or pancreatic necrosis.^1^ Wandering ascarids occasionally pass from the nose or the mouth during vomiting.

**Figure 1.**
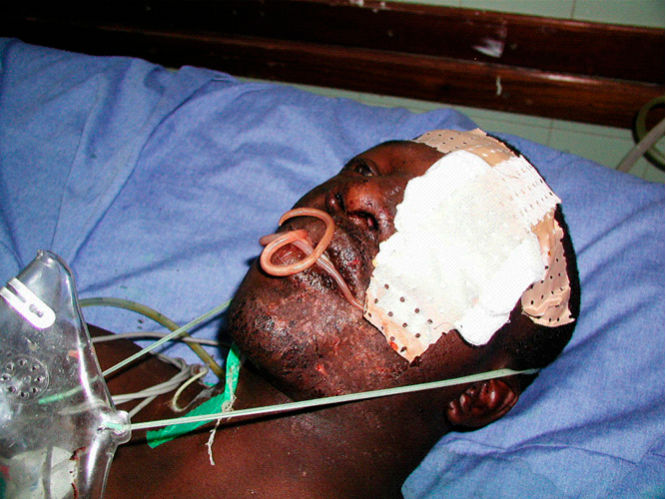
Adult Ascaris worm exiting the mouth.

We report the case of a 36-year-old Senegalese man admitted to the emergency room after being hit by a bus in Dakar. He had an orbital hematoma and rib fractures. The patient was admitted overnight for observation, and had frontal pain with nausea, vomiting, and mild confusion. A female adult *Ascaris* worm was observed to exit the mouth (Figure 1). A computed tomography scan revealed a subfrontal extradural hematoma, successfully managed by the local neurosurgical team. At discharge, the patient was treated with mebendazole.
